# Assistive Robots for Patients With Amyotrophic Lateral Sclerosis: Exploratory Task-Based Evaluation Study With an Early-Stage Demonstrator

**DOI:** 10.2196/35304

**Published:** 2022-08-23

**Authors:** Robert Klebbe, Stefan Scherzinger, Cornelia Eicher

**Affiliations:** 1 Department of Geriatrics and Medical Gerontology Charité – Universitätsmedizin Berlin, corporate member of Freie Universität Berlin and Humboldt-Universität zu Berlin Berlin Germany; 2 FZI Research Center for Information Technology Karlsruhe Germany

**Keywords:** amyotrophic lateral sclerosis, disability, disabled, disabilities, assistive robotics, human robot interaction, robotic manipulator, semi-autonomous control, motor independence, activity of daily living, daily need, everyday activities, activities of daily living, development, usability, user design, motor impairment, physical disability, robot, assistive technology, assistive device, Europe

## Abstract

**Background:**

Although robotic manipulators have great potential in promoting motor independence of people with motor impairments, only few systems are currently commercially available. In addition to technical, economic, and normative barriers, a key challenge for their distribution is the current lack of evidence regarding their usefulness, acceptance, and user-specific requirements.

**Objective:**

Against this background, a semiautonomous robot system was developed in the research and development project, robot-assisted services for individual and resource-oriented intensive and palliative care of people with amyotrophic lateral sclerosis (ROBINA), to support people with amyotrophic lateral sclerosis (ALS) in various everyday activities.

**Methods:**

The developed early-stage demonstrator was evaluated in a task-based laboratory study of 11 patients with ALS. On the basis of a multimethod design consisting of standardized questionnaires, open-ended questions, and observation protocols, participants were asked about its relevance to everyday life, usability, and design requirements.

**Results:**

Most participants considered the system to provide relevant support within the test scenarios and for their everyday life. On the basis of the System Usability Scale, the overall usability of the *robot-assisted services for individual and resource-oriented intensive and palliative care of people with ALS* system was rated as excellent, with a median of 90 (IQR 75-95) points. Moreover, 3 central areas of requirements for the development of semiautonomous robotic manipulators were identified and discussed: requirements for semiautonomous human-robot collaboration, requirements for user interfaces, and requirements for the adaptation of robotic capabilities regarding everyday life.

**Conclusions:**

Robotic manipulators can contribute to increase the autonomy of people with ALS. A key issue for future studies is how the existing ability level and the required robotic capabilities can be balanced to ensure both high user satisfaction and effective and efficient task performance.

## Introduction

### Background

Amyotrophic lateral sclerosis (ALS) belongs to the group of motor neuron diseases and is a chronic degenerative disease of the motor nervous system. Recent data indicate incidence of 0.6 to 3.8 and prevalence of 4.1 to 10.5 per 100,000 persons worldwide [1-3]. The average age of onset is between 58 and 63 years [4,5], with the youngest patients being aged between 20 and 30 years [1,6]. The male-to-female ratio shows a slightly high chance for men to develop the disease [6,7]. During the course of the disease, there is progressive loss of voluntary motor function, leading up to complete paralysis [5]. Leading symptoms of the disease include progressive muscle paresis, muscle atrophy, and muscle spasticity; however, body and sensory perception are not affected. The disease initially begins in an isolated muscle region and progressively spreads from there. The continuous loss of motor function owing to the disease leads to multiple limitations in manipulative abilities related to activities of daily living (ADLs), leading to high dependence and need for support in those affected. The corresponding support network usually consists of professional and informal caregivers who share the burden of the need to provide the necessary assistance and, at the same time, respecting and promoting the independence and self-determination of those affected [8-10]. In this context, assistive technologies and devices play a prominent role in the disease management among users who are affected [11]. According to the American Assistive Technology Act of 2004, assistive technologies and devices are defined as “...any item, device, or product system, whether commercially purchased, modified, or customized, that is designed to increase, maintain, or improve the functional abilities of individuals with disabilities” [12]. Currently, various assistive technology systems are in use to compensate for the loss of body function (eg, life support devices such as ventilators and feeding tubes, environmental control devices, orthotics, transfer devices, augmentative and alternative communication devices, and mobility aids such as powered and manual wheelchairs) [11,13]. However, in general, many of these technologies are highly specialized (task-limited assistive devices), with clearly defined and often nonmanipulative functional applications. ADLs, such as picking up and placing objects independently, preparing food, eating, and drinking, or independent personal hygiene can be addressed by these systems only to a limited extent, if at all. In this context, the use of assistive robotic manipulators is expected to have great potential in promoting independence and motor self-determination among people with functional limitations. Despite a high demand for assistive robotic manipulators in the target groups, currently, only a few systems are commercially available, and only a small proportion of those affected are provided with such systems. Reasons for this include technical, economic, and normative challenges and insufficient system implementation potential into existing care processes [14]. In contrast, there is low level of empirical evidence on the perceived usefulness and acceptance of the systems by the potential user groups [15,16]. The following section provides an overview of the current state of the art.

### State of the Art

Research and development on assistive robotic manipulators to assist people with functional limitations dates back to the 1960s [16,17]. The key functionality of such manipulators is to promote the user’s independence by compensating for functional limitations, especially with respect to the upper limbs. Driessen [18], who refers to robotic manipulators as rehabilitation robotic devices, divides them into three categories: (1) single-task robots, (2) workstations, and (3) wheelchair-mounted manipulators. Single-task robots are specialized to perform a specific task that is implemented as a predefined operational sequence in the robot controls and, as a result, can be retrieved using very simple input devices. Examples of commercially available single-task robots include various food intake assistance systems such as My Spoon (Secom), obi (Design LLC), and Bestic (CaminoCare). These systems provide a robotic arm with a spoon (in some cases, also a special plate for portioning the meal) and a simple interaction interface, which can be extended in most cases using individually designed controls. However, the potential for promoting independence is relatively low for single-task robots owing to the high degree of specialization and the required standardization of the operational environment. In contrast, robotic lightweight arms are used as stationary workstations or as manipulation aids attached to a wheelchair. Stationary workstations allow the user to detect various objects in a predefined manipulation area and to have the robotic manipulator pick them up and position them using predefined functions. Therefore, workstation systems have high flexibility with respect to the manipulation tasks, but remain limited to a fixed location. Wheelchair-mounted manipulators form the last category. These robot arms provide 6 df (7 df including the gripper) and are characterized by very slim and lightweight design [16,18-20]. Well-known and commercially available assistive robot arms for assisting people with mobility impairment are Manus, iARM (Exact Dynamics), and JACO and MICO (Kinova). These systems also have various mounting options that allow stationary use at a table or bed [19]. In addition, studies are investigating several other existing systems from the industrial setting and various prototypes; however, they have not entered the health care market [16]. Wheelchair-mounted manipulators can be used in various settings for different manipulation tasks.

The control of the systems is performed as teleoperation via a 3-axis joystick attached to the armrest of the wheelchair. The 3 df are thereby mapped to a subset of the Cartesian arm translation and wrist rotation control. To control the 7 df (3 df for movement in 3D space, 3 df for wrist movement, and 1 df for opening and closing gripper), the user must switch between different Cartesian levels [16,20].

Teleoperation by the user without specific autonomous behavior forms a control strategy that is less expensive as the user remains in charge, which reduces the complexity of the control system. Moreover, this approach offers high level of personal safety for the operation of the systems in highly unstructured and dynamic environments and in the immediate proximity of the user. In contrast, this control strategy is associated with high cognitive and physical efforts for people with physical impairments [16,18].

Against this background, current studies are investigating novel approaches to simplify the control system. The focus is on novel user interfaces (UIs) and the different possibilities of sensor fusion techniques for semiautonomous control [18,21]. In this context, Petrich et al [16] cite approaches in which participants use gestures or eye gazes to select objects that can be approached autonomously by the robot. Other interfaces for controlling robot behavior also involve electromyography, electroencephalography, and electrocorticography. As part of a systematic review of current approaches for the use of computer vision for semiautonomous control of robotic manipulators, Bengtson et al [21] highlighted three major challenges: (1) the need for adaptive semiautonomous control schemes that allow the user some control over the entire task process, (2) the handling of arbitrary objects through approaches that rely on specific grasping points and primitive shapes instead of predefined objects, and finally, (3) the precise sensing of the environment by considering different viewpoints.

In addition to the development of novel control approaches, the identification of relevant application areas in the everyday life of users who are affected is another field of research. The goal is to determine user-specific requirements for performance parameters to develop appropriate manipulation taxonomies [17,19,22].

### Robot-Assisted Services for Individual and Resource-Oriented Intensive and Palliative Care of People With ALS

Robot-assisted services for individual and resource-oriented intensive and palliative care of people with ALS (ROBINA) is a research and development project funded by the German Federal Ministry of Education and Research. The aim of the project was to develop a semiautomatic robotic manipulator that can be controlled via a multimodal UI to support people with ALS in their independence in various ADLs. Related to this, another objective of the project was to relieve professional and informal caregivers from repetitive support activities.

### Objectives

This paper summarizes the results of the final evaluation of an early-stage demonstrator developed within our project. The objective of the study was to identify the specific needs, preferences, and requirements of people with ALS for the development of a semiautonomous robotic manipulator to promote autonomy and independence in ADLs.

## Methods

### Overview

This investigation was conducted as an exploratory task-based laboratory evaluation study. Data collection was based on a mixed methods design comprising validated and self-developed questionnaires, standardized observation protocols, and semistructured interviews. The study was designed as a task-based evaluation, and the study duration was 2 weeks.

### Laboratory Study Setting

**Figure 1:**
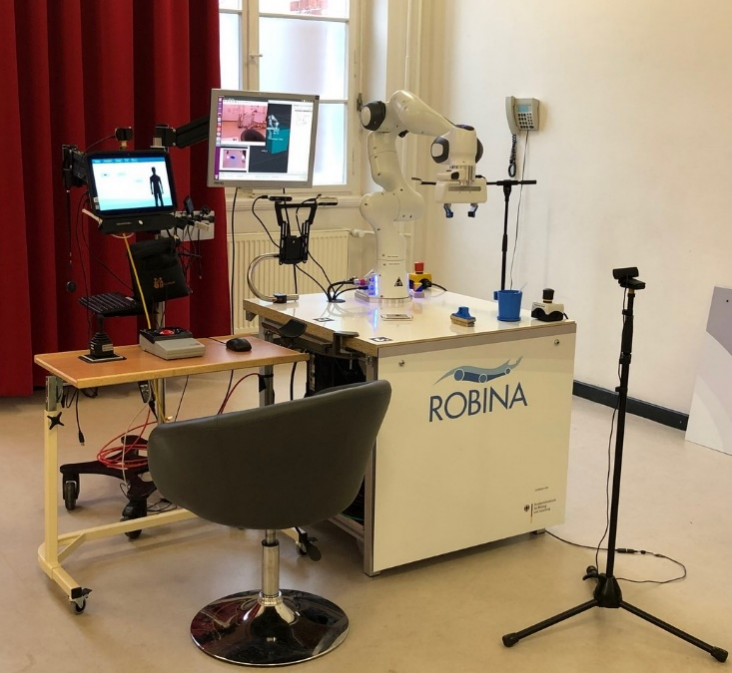
Evaluation setup of the robot-assisted services for individual and resource-oriented intensive and palliative care of people with amyotrophic lateral sclerosis system.

Figure 1 shows the setup for the study. It was built around a 7-axis Panda manipulator (Franka Emika) with torque sensors in its joints that enabled it to interact sensitively with the participants. Moreover, the robotic system was equipped with a 2-fingered gripper. The functional modes of the gripper included opening and closing of the 2 fingers. Rotation of the gripper to align it with the manipulation object was not possible. The control software was based on the “Robot Operating System” [23], a software framework established in robotics research for developing complex applications. It combined open-source components and custom-developed enhancements into a state machine that managed the patients’ inputs and overall control flow. The robot was controlled over the provided Franka control interface that enabled a real-time bidirectional communication. Custom-built Robot Operating System controllers used and regulated the robot’s capabilities to mimic the physical appearance of a mechanical spring. The software ran as a distributed system on 3 PCs that communicated over a shared, closed network. In total, 2 of the PCs performed computationally intensive operations with real-time communication to the robot and red, green, blue, and depth (RGB-D) camera-based object detection in a Linux-based operating system (Ubuntu Desktop 16.04 Long Term Support; Canonical Foundation, Ubuntu Community). The software for control via the patient’s sensors ran on a tablet with Windows operating system. It was implemented as a locally communicating application for modern internet browsers and accessed via control units by the patients. These control units comprised a variety of input devices to best cover each participant’s capabilities, such as joysticks that were directly operated in the hand or attached to a gooseneck mount, a head control system (Smart Nav Natural Point), and an eye control system (Alea Technologies gmbh) that offered control when only eye gaze was available.

**Figure 2:**
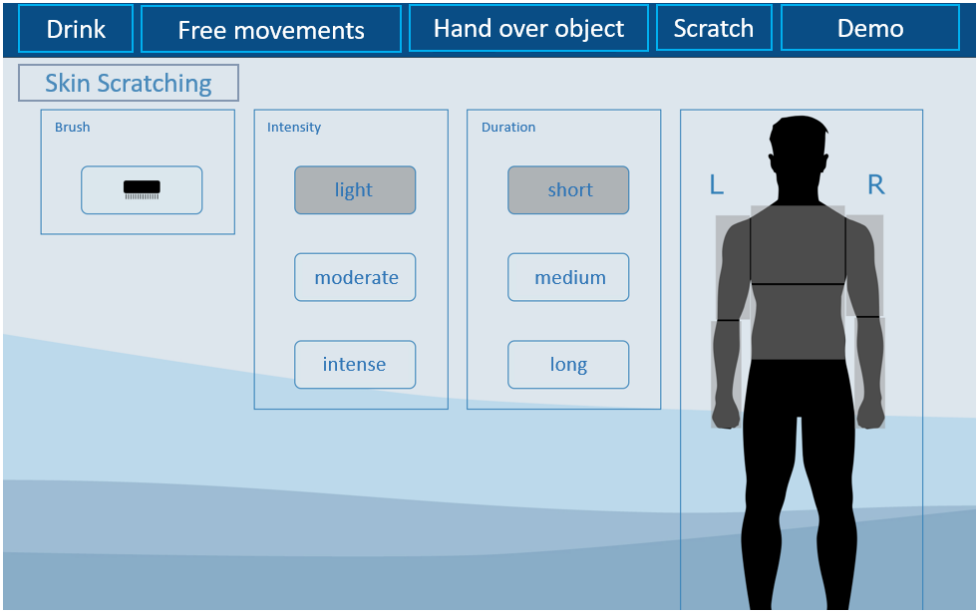
Graphical user interface for the scratching scenario, with customizable settings.

**Figure 3:**
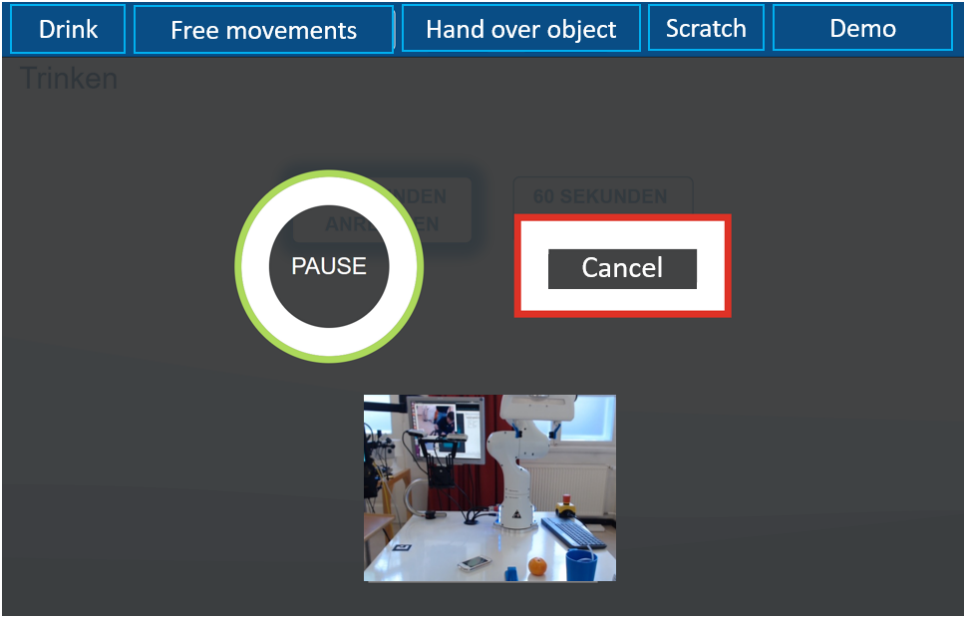
Graphical user interface during the execution of a robot action, with the options to pause or cancel the execution by the user themselves.

All inputs were mapped to a mouse pointer, with which the patients navigated the menus of the browser-based graphical user input (GUI) and controlled the robot. Different task scenarios (refer to the following section) were implemented as movement sequences that the participants could execute, pause, reset, and customize to their needs by adjusting the parameters of the workflow (Figures 1-3). The system supported partial autonomy, such as face and lip detection during drinking and visual-based grasping of objects from a tabletop.

### Evaluation Tasks

The different task scenarios for the evaluation are described in the following sections.

#### Serve a Drink

The robot system serves a cup with a silicone straw. After selecting the requested function via the UI, the robotic manipulator grips the cup autonomously with its 2-fingered gripper. The movement to the user’s mouth is determined from the calculated pose for the center of the mouth in relation to the tip of the straw. The robot autonomously leads the cup up to 10 cm from the mouth of the participant, by using visual mouth tracking. To drink, the participant must actively move their head toward the straw.

#### Hand Over a Mobile Phone

The study participant initiates the *hand over of a mobile phone* by clicking on the corresponding icon on the UI. Then, the robot system picks up and places the mobile phone autonomously in a predefined transfer zone. The phone is in a predefined pickup area, and the robot grasps the mobile phone autonomously with its end effector. It tracks the phone via visual object recognition.

#### Skin Scratching

The participant initiates the task by choosing the duration and intensity of scratching and the type of brush (Figure 2). The robot arm autonomously picks up the brush and then slowly approaches the human forearm. The forearm of the participant rests on an arm padding, which serves the robot system as position recognition. The robot sensors continuously check the contact between the brush and the human arm to adjust the robot movement in case of limb position changes. If contact is interrupted, time limit is exceeded, or execution is stopped by the user, the robot stops scratching, places the brush back on the table, and returns to its standby position.

#### Free Manipulation

In this task, the study participant can move the robot arm freely in a defined area and manipulate objects. For a standardized assessment of the task, the participants were asked to stack cubes on top of each other. The participant controlled all movements of the robot systems by clicking six direction levels (left, right, up, down, backward, and forward) and opening and closing the gripper on the UI.

### Participants’ Safety

Owing to the early-stage demonstrator status of the ROBINA system, risk analysis was conducted, defining necessary measures for participants’ safety. Principal measures included conducting the evaluation under laboratory conditions and technical supervision by specially trained staff. Furthermore, except for the *scratching* scenario, the robotic arm could not reach the study participant at any time. In addition, formal and informal caregivers of the participants were included in the study to assist in the monitoring of their well-being and general condition. Moreover, a familiarization phase for the ROBINA system and evaluation task was conducted at the beginning. Another safety measure was a “Pause/Cancel” button on the UI, with which the participants could interrupt each scenario at any time. In addition, the correct execution was monitored by specially trained staff and could be interrupted by them immediately. All materials used were checked for sharp edges or damages, and participants were required to wear safety goggles throughout the testing procedure.

### Ethics Approval

The ethics committee of the Charité – Universitätsmedizin Berlin, corporate member of Freie Universität Berlin and Humboldt-Universität zu Berlin approved the study (EA2/145/19). Moreover, the study was registered with the German Clinical Trials Register (DRKS00016554).

### Study Population

Participants were recruited by the Geriatrics Research Group over a period of 4 weeks. The following were the inclusion criteria for the study: participants were aged ≥18 years and had clinically diagnosed ALS. To investigate the functional limitations that influence the operation of the ROBINA system, we used the ALS functional rating scale (ALS-FRS) [24], limited to the two questionnaire dimensions on limitations in speech and finger function.

In total, 11 individuals with clinically diagnosed ALS participated in the study. Of the 11 participants, 8 (73%) were male and 3 (27%) were female. The mean age was 57.1 (SD 5.9; range 51-70) years. In total, 73% (8/11) of the participants had their arms affected (arm paresis, tetraparesis, or similar conditions). Regarding the ALS-FRS dimension regarding speech, of the 11 participants, 4 (36%) participants showed no limitations, 5 (45%) showed mild to medium limitations, and 1 (9%) had lost the ability to speak. Regarding functional limitations of the fingers, of the 11 participants, 1 (9%) participant stated that they have no limitations, 6 (55%) mentioned mild to medium limitations, and 4 (36%) have severe limitation (ie, they were not able to press keys on a keyboard).

### Study Procedure

Participants were contacted first via telephone and informed about the purpose and procedure of the study. After providing formal consent, they were invited to the research facility of the Geriatric Research Group.

As a first step, sociodemographic data and subdomains of ALS-FRS-Extended were recorded. Then, the most appropriate control device for operating the research demonstrator was selected with the assistance of an experienced project partner and set up according to the participant’s needs (eg, head control, eye control, joystick, PC mouse, or ball mouse). In the second step, familiarization with the system and test scenarios was conducted. In this context, the experimental setup and procedure of the single scenarios, UI, robot actions, and required safety measures were presented. In addition, a functional demonstration of the system was performed to familiarize the users with the system.

Subsequently, the task-based evaluation phase was conducted, in which the study participants tested and evaluated each of the scenarios presented in the previous sections. During the execution, a standardized observation protocol was used to record the system and user errors and spontaneous expressions of the participants (think aloud). In addition, after each task, participants were asked to rate the system using a self-developed, standardized, and validated questionnaire (refer to the following sections).

### Quantitative Evaluation

On the basis of a self-developed questionnaire, participants in the task-based intervention section of the study were asked to rate the categories of relevance to everyday life, usability, and feeling of safety during task execution for each scenario on a 5-point Likert scale. Another item about the preference for human support over robotic support comprised 3 response categories. The questions asked under each category are shown in Table 1.

**Table 1:** Self-developed questionnaire to evaluate the robot-assisted services for individual and resource-oriented intensive and palliative care of people with amyotrophic lateral sclerosis system regarding usability.

Categories and questions		Response categories
**Relevance to everyday life**		
	How relevant do you think the scenario is to your current everyday life?	1=very relevant to 5=not relevant
**Usability**		
	How do you rate the operability with the control unit you use?	1=very easy to 5=very difficult
	How did you feel about the speed of movement of the robotic manipulator?	1=very fast to 5=very slow
	How did you feel about being [e.g., served a drink or scratched] by a robotic manipulator?	1=very comfortable to 5=very unpleasant
**Feeling of safety during task execution**		
	How safe did you feel during the execution of actions in the...scenario?	1=very safe to 5=very unsafe
**Preference for human assistance**		
	In the current scenario, would you prefer the assistance of a human to that of the robotic manipulator?	1=yes, 2=no, and 3=do not know

The general evaluation of the ROBINA system was based on the System Usability Scale (SUS) [25], which is a simple and technology-independent instrument for assessing the subjectively perceived usability of a technical system. The SUS comprises 10 items that are answered on a 5-point Likert scale. The answers of the users are transformed according to a recoding table and then summed up (percentile interpretation). The possible score ranges from 0 to 100 points, whereby a score of 68 is required as a benchmark for at least good usability. A score of 100 corresponds to perfect usability.

In addition to SUS, a self-developed questionnaire was used to determine user perception in the following categories: feelings of anxiety during use, system size, and design of the graphical UI (Table 2).

**Table 2:** Self-developed questionnaire to evaluate the robot-assisted services for individual and resource-oriented intensive and palliative care of people with amyotrophic lateral sclerosis system regarding user perception.

Categories and questions			Response categories
**Feeling of anxiety**			
	Have you been afraid during the testing of the robotic manipulator?	1=great fear, 2=little fear, and 3=no fear	
**System size**			
	How did you feel about the size of the robot manipulator?	1=too big, 2=appropriate, and 3=too small	
**Design of the graphical user interface**			
	How did you like the design of the graphical user interface?	1=very good to 4=very poor	
	How well could the elements be recognized on the graphical user interface?	1=very good to 4=very poor	
	How well did the robot performance meet your expectations towards task description in the graphical user interface?	1=very good to 4=very poor	

### Qualitative Evaluation

To gain deep insight into the subjective perception and evaluation of the ROBINA system, qualitative data were collected in 2 ways. First, from open-ended questions—regarding task-based evaluation, the participants were asked to state their preference for human or robotic assistance. In addition, they were asked about the aspects of each scenario that they like the most and those that they do not like at all. In the general evaluation, the participants were asked an open-ended question about the suggestions they had for improving the UI. Second, qualitative data were collected using observation protocols. As part of a think-aloud protocol, participants’ spontaneous expressions during testing were recorded; human and technical errors were also recorded.

### Analysis

Quantitative data were analyzed descriptively using SPSS (version 28.0; IBM Corp) for Windows. Results are presented as medians, IQRs, and minimums and maximums as most of our data had an ordinal scale level or did not have a Gaussian normal distribution. Owing to the exploratory nature of our laboratory study, hypotheses and significance tests were not performed.

Qualitative results obtained from open-ended questions and observation protocols were analyzed using systematic structuring content analysis, according to Mayring [26]. Considering the targeted study objective, the analysis included the paraphrasing of content-relevant text passages in the different materials. On this basis, the targeted level of abstraction was determined, and the paraphrases were generalized under that level. Subsequently, the first reduction of paraphrases with the same meaning was conducted through selection. In a further reduction step, paraphrases were pooled and integrated at the targeted abstraction level. To ensure data quality, these analysis steps were conducted by 2 trained researchers, who have experience with qualitative studies. The analysis steps of paraphrasing, generalization, and reduction were performed using Excel (version 2016; Microsoft).

## Results

As mentioned in the previous section, the experimental setup included various input devices to best meet the abilities of each participant. The following input devices were used to control the ROBINA system via the GUI within the four scenarios: eye control (1/11, 9%), normal PC mouse (3/11, 27%), ball mouse with switch (1/11, 9%), head control (5/11, 45%), and wheelchair joystick (1/11, 9%).

### Task-Based Evaluation

**Figure 4:**
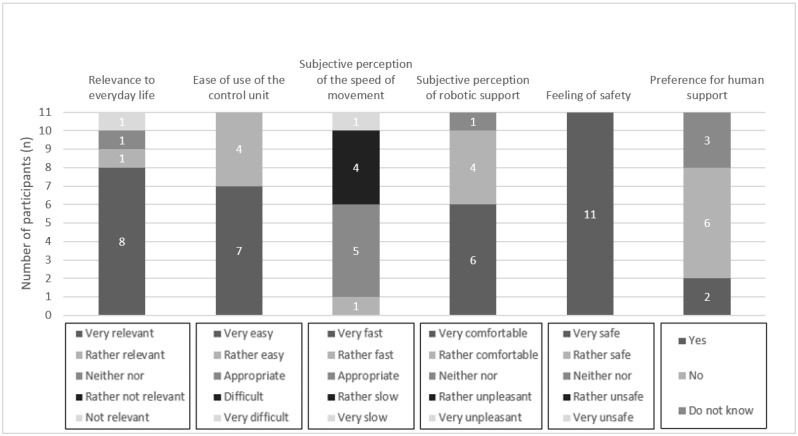
Aggregated presentation of the response distributions in the categories’ relevance of the evaluation scenario (serve a drink) to everyday life, usability, feeling of safety during semiautonomous robotic behavior, and preference for human assistance over robotic assistance.

**Figure 5:**
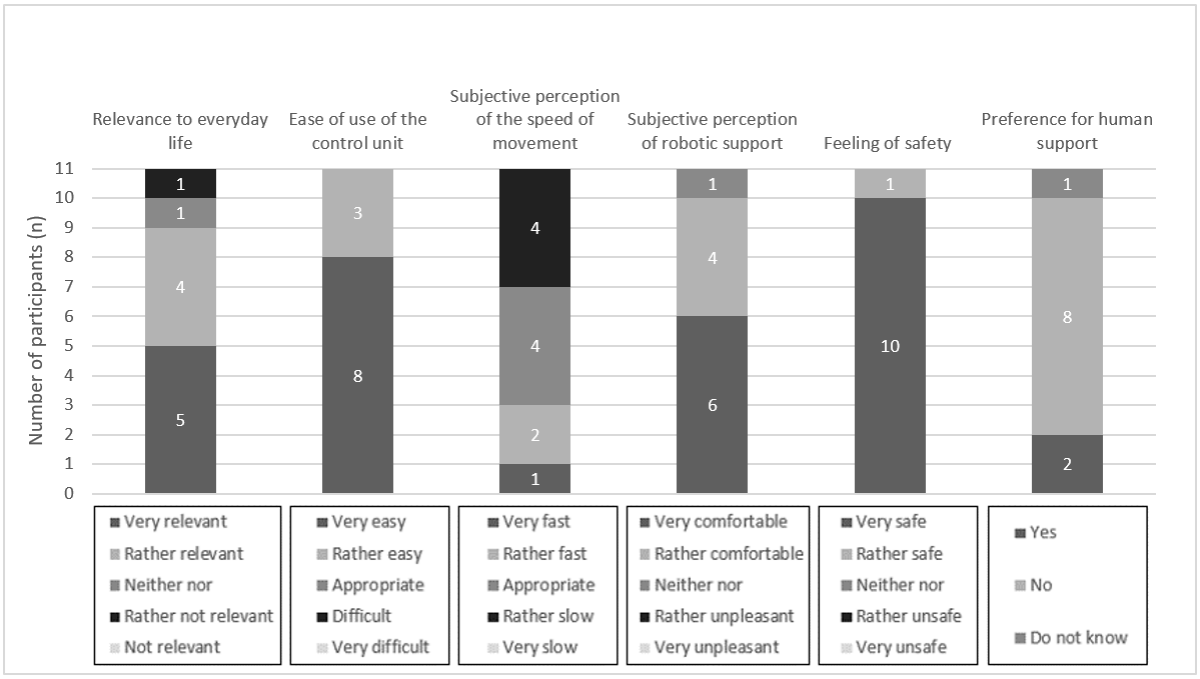
Aggregated presentation of the response distributions in the categories’ relevance of the evaluation scenario (hand over a mobile phone) to everyday life, usability, feeling of safety during semiautonomous robotic behavior, and preference for human assistance over robotic assistance.

**Figure 6:**
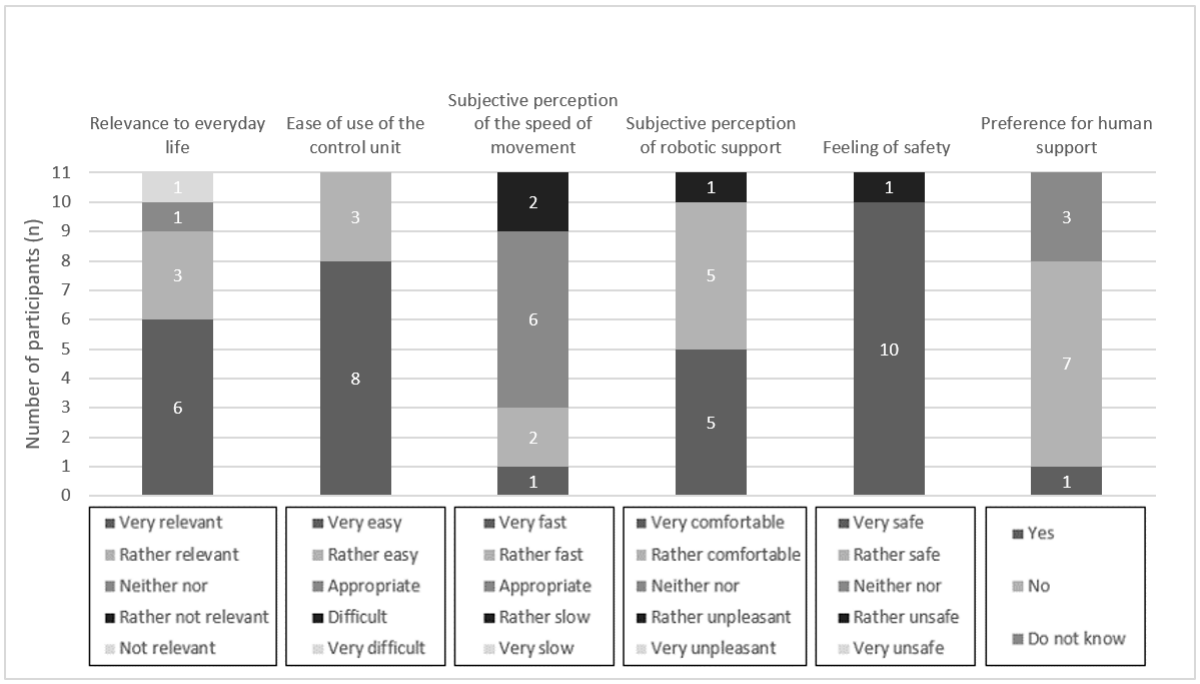
Aggregated presentation of the response distributions in the categories’ relevance of the evaluation scenario (scratching) to everyday life, usability, feeling of safety during semiautonomous robotic behavior, and preference for human assistance over robotic assistance.

**Figure 7:**
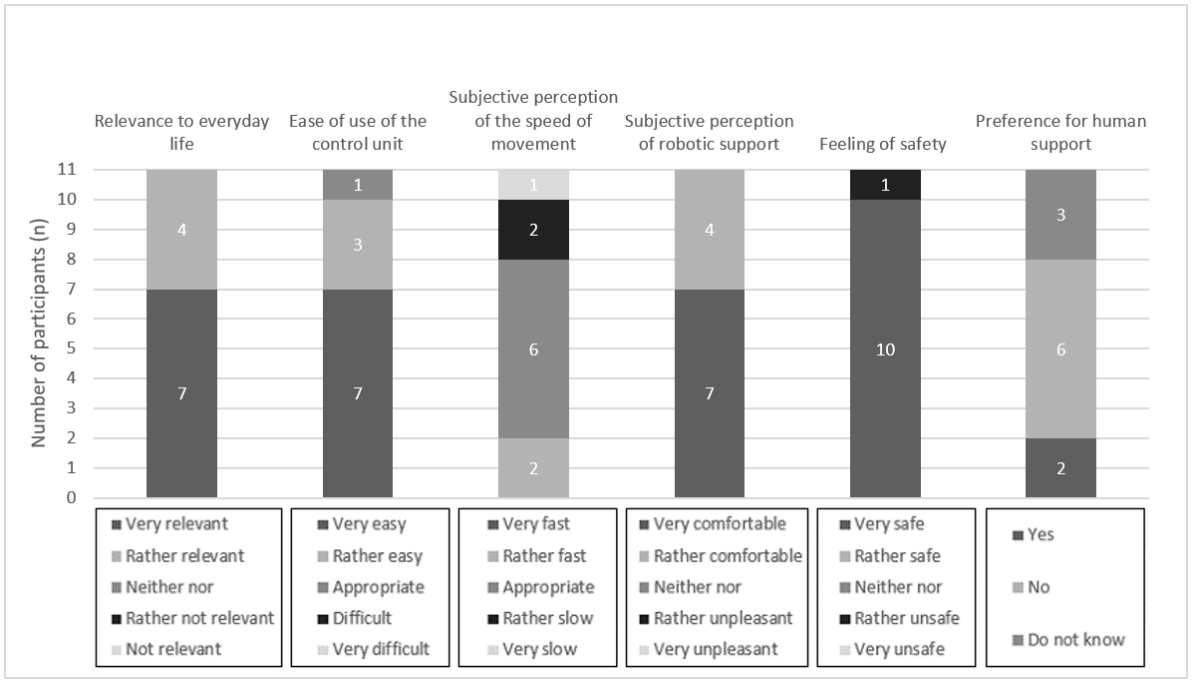
Aggregated presentation of the response distributions in the categories’ relevance of the evaluation scenario (free movement) to everyday life, usability, feeling of safety during semiautonomous robotic behavior, and preference for human assistance over robotic assistance.

In the following section, the results of the task-based evaluation of the ROBINA system are presented. In this context, the relevance of the evaluation scenario for the everyday life of the participants who are affected, usability (including ease of use of the control unit, speed of semiautonomous robotic movement, and subjective perception of the robotic support), feeling of safety, and preference for human support over robotic support are described. For better illustration of the results, they are also presented graphically (Figures 4-7). Finally, the presentation of each scenario ends with the participants’ assessments obtained from the open-ended questions.

Most participants (9/11, 82%) rated the relevance of the *serve a drink* scenario for everyday life as very or rather relevant. Only 9% (1/11) of the participants rated this scenario as not relevant for everyday life. Regarding usability, all participants (11/11, 100%) rated the ease of use of the control unit as very easy or rather easy. The movement speed of the robot was rated as appropriate by 45% (5/11) of the participants. In contrast, 45% (5/11) of the participants rated it as rather slow or very slow. A participant perceived the movement speed to be rather fast. The subjective perception of the robotic support in this scenario was rated as very comfortable or rather comfortable by most participants (10/11, 91%). A participant perceived it to be neither pleasant nor unpleasant.

Furthermore, all participants (11/11, 100%) stated that they felt very safe while performing the semiautonomous robotic behaviors.

Finally, for the *serve a drink* scenario, 18% (2/11) of the participants indicated that they would prefer human assistance. In contrast, more than half of the participants (6/11, 55%) indicated that they would not prefer human assistance. Of the 11 participants, 3 (27%) participants could not provide a preference.

As part of the qualitative evaluation, the participants were asked about the aspects of the *serve a drink* scenario that they particularly liked or disliked. In particular, the participants appreciated the precise and fast reactions and the smooth motion. Moreover, the semiautonomous actions were mentioned positively. However, at the same time, participants preferred to fully control the system as long as they were physically and cognitively able to do so. In the event of a physical or cognitive decrease, for example, owing to fatigue, participants preferred the system to take over control and act autonomously. Offering a drink to the mouth was found to be pleasant and a great relief. The size of the system, which makes it unsuitable for home use, was critically highlighted. In addition, a respondent criticized the system for taking different paths to pick up and serve the cup. In another case, the cup was served in a slightly skewed position; thus, the risk of spilling liquid was criticized. In 3 cases, the system collided with the surrounding devices when returning to the starting position (twice with the tablet and once with a wheelchair control), which caused irritation among the participants. Of the 11 participants, 2 (18%) participants noted the incompleteness of the scenario, as a third person was required to fill the cup and bring it into the robot’s interaction field.

A key requirement that emerged from the *serve a drink* scenario was the reliability of the robot’s actions in an unstructured environment and when manipulating objects. In this context, a participant stated that the robot needed to know its interaction radius. For reliable object manipulation, the system should also be able to recognize the material of the objects and grasp them with appropriate force. A third finger was suggested to increase the reliability of grasping.

The *hand over a mobile phone* scenario was rated as very relevant or rather relevant for their everyday lives by most respondents (9/11, 82%). A participant rated it as neither relevant nor irrelevant, and another participant rated it as rather not relevant.

Regarding the 3 questions on the usability of the ROBINA system in this scenario, the ease of use of the control unit was rated as very easy or rather easy by all participants (11/11, 100%). The evaluation of the speed of movement showed a differentiated image. Of the 11 participants, 3 (27%) participants rated it as very fast or rather fast and 4 (36%) other participants rated the movement speed as adequate. Similarly, 36% (4/11) of the participants rated the speed of movement as rather slow. Finally, the robotic assistance was rated as very comfortable or rather comfortable by most respondents (10/11, 91%). A participant perceived it as neither pleasant nor unpleasant.

Regarding participants’ feeling of safety during the semiautonomous task execution, all participants indicated that they felt very safe or rather safe (11/11, 100%).

Similar to the *serve a drink* scenario, 18% (2/11) of the respondents indicated a preference for human assistance. In contrast, 73% (8/11) of the participants did not prefer human assistance over robot assistance. A participant could not report a preference.

Regarding qualitative evaluation, the transfer of objects into the interaction field of the individuals who are affected, careful pickup of the mobile phone, and fast and precise motion sequence over large distances were described as positive. In general, the task was described as being “close to reality.” However, in a few cases, the phone was dropped rather than carefully put down during the delivery. In addition, participants emphasized that the scenario was only suitable for people who can still pick up and operate a phone independently.

In summary, 3 key requirements were mentioned: first, direct transfer of the mobile phone to the user or into an appropriate holder; second, connection of the mobile phone control to the robot or wheelchair control (to operate it); and third, safety function that prevents the robotic system from dropping an object during the transfer.

In total, 82% (9/11) of the participants rated the *scratching* scenario as very relevant or rather relevant for their everyday life. Of the 11 participants, 1 (9%) participant each rated it as neither relevant nor irrelevant.

The ease of use of the control unit was rated as very easy or rather easy by all participants (11/11, 100%). In the *scratching* scenario, the evaluation of the speed of movement varied. Of the 11 participants, 3 (27%) participants found the speed to be very fast or rather fast, approximately half of the participants (n=6, 55%) rated it as appropriate, and 2 (18%) participants felt the speed was rather slow. Robotic assistance was rated as very comfortable or rather comfortable by most respondents (10/11, 91%). A participant perceived it as rather unpleasant.

Most participants (10/11, 91%) felt very safe during the semiautonomous task execution. A participant rated the feeling of safety as rather unsafe.

Preference for human assistance over that provided by the robot was not expressed by most participants in the *scratching* scenario (7/11, 64%). However, a participant indicated preference for human assistance. In total, 27% (3/11) of the participants were not able to provide a preference.

In the qualitative evaluation of the *scratching* scenario, a participant particularly liked the quick satisfaction of solving an acute problem and the increase in privacy and independence. Moreover, participants perceived the scratching as pleasant, however, depending on the brush and skin type. Similarly, the degree of scratching duration and intensity explicitly corresponded to the ideas of the users, as did the possibility to adjust them. In contrast, a participant questioned the practicality of the task, particularly in the facial area. Another user was unsure how the system localized the area to be scratched. Uncertainty among participants occurred in cases where the system picked up the brush, skewed with the 2-finger grippers. In these cases, participants expressed concern about injury to the skin. The requirements for the correct positioning of the participant in relation to the ROBINA system were also viewed critically, because although this was plausible for safety reasons, it could not be implemented in everyday life independently by patients with ALS and limited mobility. In this respect, dependence on other people will remain. It was further critically stated that the positioning of the participant in relation to the system results in an irregular scratching movement, and therefore, the intensity varies over the distance of the scratching movement. Finally, the lack of a separate start button in the GUI was criticized, as it was not clear to the participants how the scenario can be started once the parameters had been selected.

As requirements for further development in this scenario, the possibility of the exact determination of the location of the itch instead of the vague selection of whole-body regions was highlighted. Another requirement was a clearly defined button to start the scenario. Finally, some participants wished for better adaptation of the scratching movements and the brush to the body shape.

All participants (11/11, 100%) rated the relevance of the *free movement* scenario as very relevant or rather relevant for their everyday life.

Regarding usability, the ease of use of the control unit was rated by most participants (10/11, 91%) as very simple or rather simple. A participant evaluated it as adequate. The speed of movement was also considered differently in this scenario. Of the 11 participants, 2 (18%) participants perceived it as rather fast, more than half of the participants (n=6, 55%) found it to be adequate, and 3 (27%) participants rated it as rather slow or very slow. The robotic support was rated as very pleasant or rather pleasant by all participants (11/11, 100%).

The subjective feeling of safety during the robotic executions was rated as very safe by 91% (10/11) of the participants. A participant reported to have felt rather unsafe.

For this scenario, of the 11 participants, 2 (18%) participants preferred human support, 6 (55%) other participants did not prefer human assistance to that provided by the robot, and 3 (27%) participants could not indicate a preference.

In the qualitative evaluation of the *free movement* scenario, the perceived independence from human assistance was highlighted. A participant mentioned that he would prefer care assistant to the system. However, if verbal communication was no longer possible for him, this task would be of great importance. In addition, both the precise movement control (ability to choose between small and large movements) and the sensitivity of the ROBINA system were positively highlighted. Critically, in this scenario, it was emphasized that the movement speed could not be adjusted. Furthermore, regarding the use of head control, it was emphasized that holding the head position and the many micromovements to trigger robot movements were strenuous. Another point of criticism was the nonuniformity of the robot’s movements, which did not follow a straight line.

As requirements for further development, several users wished for stepless control. For movements over long distances, a context menu that will allow speed control via sliders was suggested. Alternatively, movements over long distances can depend on the duration of pressing a corresponding button on the graphical UI.

Across the scenarios, 36% (4/11) of the participants described a potential for the promotion of independence and autonomy by the system. In total, 18% (2/11) of the participants had no preference for human or robotic support. Another 18% (2/11) of the participants stated that they will only use robotic assistance as long as it did not lead to the total replacement of their caregivers. A participant felt more comfortable with humans. This participant stated that they will use robotic assistance if their physical functionalities were very limited or if no other person was present to provide support. Apart from that, the respondent perceived the robot as a burden relief for his relatives.

### General Evaluation

Following the task-based evaluation, the participants were asked to provide a general evaluation of the system.

The overall usability of the ROBINA system was measured using SUS. On average, the ROBINA system was rated with median of 90 (mean 86.1; IQR 75-95; minimum 70; maximum 97.5) points, and thus, ranked in the upper range of “excellent” or grade A [27].

In addition, based on a self-developed questionnaire, participants were asked questions about the perceived fear while using the ROBINA system, the system’s size, and the design of the graphical UI.

For the task-based use of the ROBINA system, all participants (11/11, 100%) indicated that they had not felt any anxiety.

Regarding the size, 64% (7/11) of the participants felt that the ROBINA system was very large and 36% (4/11) of the participants felt it was appropriate.

**Figure 8:**
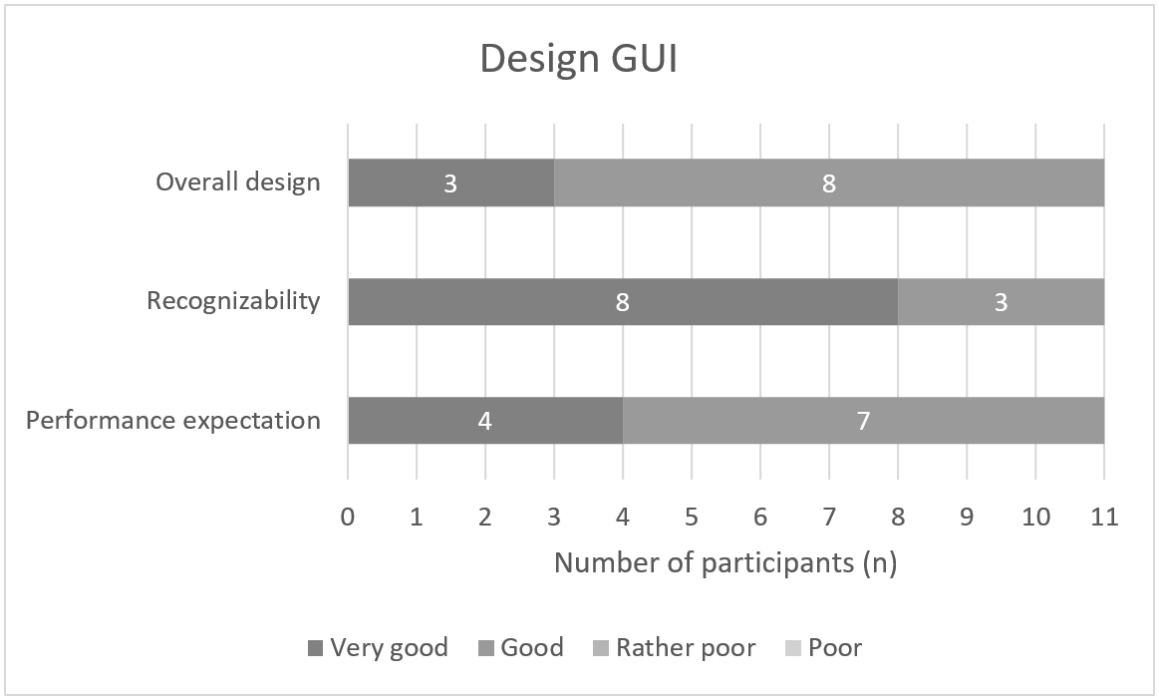
Responses to the survey questions about the general evaluation of the graphical user interface (GUI).

Another focus was the general evaluation of the graphical UI. This was generally assessed as good (8/11, 73%) or very good (3/11, 27%) by most participants. Regarding the visualization of the various functions in the graphical UI, 73% (8/11) of the participants stated that these were very well recognizable. In total, 27% (3/11) of the participants rated it as good. In addition, 64% (7/11) of the participants indicated that the representations for semiautonomous execution by the ROBINA system on the graphical UI met their expectations for robot performance well. Overall, 36% (4/11) of the participants indicated that the actual executions met these expectations very well. Figure 8 presents a graphical overview of the participants’ evaluations. Within the qualitative evaluations of the graphical UI, the participants were asked to provide detailed suggestions for improvement. According to the participants, 3D symbols should be displayed to better clarify the robot’s control directions. The font should be more legible (ie, large and thick) and contrasting to the background. Contrast and brightness should be adjusted for operation in the dark. In general, some settings such as color and contrast should be customizable. When using the head control, there was a risk that the user would unconsciously trigger a function without looking at the screen. In 2 cases, the participants actively approached the study staff about this concern, and in another case, there was actually an unconscious cancellation of the running task after the participant had averted his gaze from the tablet to the real task execution. In total, 18% (2/11) of the participants recommended an area within the control design of the GUI into which the user can look without fear of triggering something unconsciously. In the *free movements* task, users were in favor of revising the navigation label or making it more intuitive by using a suitable color concept. Furthermore, the live image of the robot’s position with respect to the manipulation object in the graphical UI was hardly used. Instead, the participants observed the process in the real study setup. The participants explained that this was because of the small size of the live image in the graphical UI, which did not show the entire interaction space of the robot. Another reason was that spatial perception of the interaction area via the 2D live image was severely limited (Figure 3).

## Discussion

### Principal Findings

This study aimed to investigate the key requirements and needs of people with ALS for the use of a semiautonomous robotic manipulator for supporting ADLs. For this purpose, four exemplary activities (*serve a drink*, *hand over a mobile phone*, *scratching*, and *free movement*) were evaluated in an explorative and task-based laboratory study with 11 individuals from the target group. The study was based on a multimethod approach comprising quantitative and qualitative methods.

Regarding the quantitative part, the use of a robotic manipulator was considered to be relevant in the investigated exemplary scenarios. Most participants evaluated the operation of the system as easy and the semiautonomous robotic actions as pleasant. At the same time, most participants felt safe during the semiautonomous robot actions. Differences existed, especially regarding the execution speed of the semiautonomous robot actions and the preference for human assistance over robotic support.

The qualitative analysis of the open-ended questions about the application and the observation and think-aloud protocols provided a deep insight into the user-centered assessments and development requirements. These findings can be summarized into 3 requirement areas.

The first area concerns the role and design of semiautonomous robot actions. In general, the investigated semiautonomous robot capabilities were evaluated positively. The precise and dynamic motion sequences and the careful picking up of objects were particularly highlighted. However, at the same time, errors such as irregular motion paths, collision with equipment in the environment, and inaccurate pickup of objects became evident. Against this background, precise and reliable execution of semiautonomous robot motions and object manipulations and environmental and object recognition capabilities were key development requirements. Another central result concerns the execution speed, which should be customizable according to the user’s abilities. Generally, the participants desire a largely self-responsible control of the robotic manipulator. In contrast, semiautonomous robotic actions should be applied when the user’s physical abilities decline during the day (eg, owing to fatigue) or owing to the progressive course of the disease.

Another area of requirements concerns the control unit. Regarding the different input devices for operating the robot arm (such as head control, mouse control, and joystick), the use of head control was perceived as strenuous for the user in the scenarios with increased input requirements. As the input device used corresponded to the physical abilities that were still available, there is great demand for the design of a UI for those affected, who can no longer use their extremities for operation. Regarding the graphical UI, an increasing number of input options requires attention to design a differentiated display for better distinction of the corresponding robotic abilities.

Finally, the last area covers the requirements for the adaptation of robotic capabilities regarding the everyday life of the target group. According to the participants, the use of a robotic manipulator will enhance their independence, autonomy, and privacy in everyday life. However, at the same time, the current state of development of the test scenarios will still make the user dependent on human assistance. Consequently, the developed robot capabilities focused only on specific subareas of the respective everyday activity and require various preliminary activities that neither the user himself nor with use of the robot can implement independently. Finally, regarding promoting autonomy and independence, it was also emphasized that the use of robotic systems should not lead to the replacement of human assistance.

### Comparison With Previous Studies

In the following sections, our findings will be discussed in comparison with previous studies. We will focus on the three central requirement areas identified through the qualitative analysis: requirements for semiautonomous human-robot collaboration, requirements for UI, and requirements for the adaptation of robotic capabilities regarding the everyday life of the target group.

#### Requirements for Semiautonomous Human-Robot Collaboration

Regarding using semiautonomous robotic manipulators to compensate for functional limitations, 3 characteristics are of particular importance: design of the control model, handling of objects, and execution speed.

In their scoping review of recent studies on using computer vision for semiautonomous control of assistive robotic manipulators, Bengtson et al [21] found that most of the studies focused on rather fixed schemes for semiautonomous control, which are based on predefined roles for the user and the system. An advantage of this distinct distribution of role models was that the user is relieved of challenging control processes and that the accountability of responsibilities between the user and the system is simplified. However, according to the authors, a disadvantage was that the user has only limited access to autonomous processes, which in turn can have a negative impact on the user experience. As a solution to this problem, the authors suggested an adaptive semiautonomous control approach that continuously involves the user in this process. With this arbitration of control, the robot control can be more strongly adapted to the user’s capabilities and thus achieve a high degree of individualization of the human-robot collaboration.

A similar conclusion was reached by Kim et al [28]. On the basis of a vision-based 6 df UCF-MANUS, the authors conducted a comparative study of two different control models (supervised autonomous operation vs manual or Cartesian operation) with the target group of individuals with traumatic spinal cord injury. The evaluation was conducted over 1 to 2 hours weekly, over a period of 3 weeks. Interestingly, both groups had comparable task completion times at the end of the study, which the authors attributed to the learning effects in the manual operating group. In addition, the authors found that the results from the autonomous operation mode showed significant reduction in the number of clicks and task completion time. At the same time, participant satisfaction did not increase. The authors concluded that participants wanted to perform the appropriate tasks independently with the robotic system. Another key finding of the study was that participants who tested both control modes required an adaptive control system that allowed them to switch between the 2 control modes as needed.

As our results have shown, another challenge of semiautonomous control models is the precise and safe object manipulation. Overall, 2 aspects are of importance here. First, the identification and localization of an object, and second, the precise and safe grasping. Regarding the first aspect, different approaches are already available, such as proximity-based approaches or the detection of an object by a sensor (such as laser pointer, eye-tracking, or electroencephalography) [21,28,29]. Regarding the second aspect of the grasping process, different approaches are currently under review, which are based either on predefined objects or specific shapes. As these approaches deal with simplified assumptions about an object, a major challenge involves the manipulation of arbitrary objects. Bengtson et al [21] considered a solution to this problem using approaches that focus either on the recognition of suitable grasping points or the decomposition of the object into different shapes. Another approach is to involve the user in identifying and marking such grasping points for the system or teach the system to grasp different shapes of everyday objects independently. Finally, another solution to improve the manipulation properties is to adapt the gripper by using at least three fingers or use specific adapters for specific objects [22,28].

The third main challenge is regarding the execution speed of robotic actions. Various studies have shown that semiautonomous control models have led to significant improvement in both success rate and execution time of tasks compared with commercially available Cartesian control models. At the same time, in accordance with our results, some studies also show that target groups desire high execution speeds [15,28]. Thus, the user-centered adaptation of movement speed can be interpreted as an essential factor for user experience. However, at the same time, this represents an essential parameter for ensuring safe human-robot collaboration. Therefore, a potential solution to this issue can be a gradual expansion of the performance level of the robotic manipulator linked to specific operating skills. This should consider both positive adaptation to the system and potential limitations of use owing to the course of the disease. Particularly considering the progressive physical decline, it currently remains unclear to what extent the users are able to perform such system configurations on their own responsibility. Thus, to support the users in their daily use of such robotic systems, an appropriate adjustment of the system configuration, especially regarding the speed of movement, should be supervised by qualified experts.

#### UI Requirements

In addition to the requirements for semiautonomous human-robot collaboration, our results show that the UI is also essential for effective and efficient use of a robotic manipulator. Currently, commercially available UIs mostly rely on teleoperation via a 3-axis joystick. The 3 df are thereby mapped to a subset of the Cartesian arm translation and wrist rotation control. To control the 7 df, the user must switch between different Cartesian levels [20]. Thus, grasping an object using a robotic manipulator is transformed into a multitude of distinct movements that require frequent switching between and within different Cartesian levels. For people with functional limitations of the upper extremities, this can result in high physical and mental stress.

In this context, Chung et al [30] investigated the performance of a tablet-based UI versus a conventional joystick control in a comparative pilot study with 8 participants with upper extremity impairments using a JACO (Kinova) robotic manipulator. The use of the touch screen UI resulted in high execution speeds and low task completion times compared with the conventional control form; however, no equal distribution of UI users was realized within the study. In addition, the participants rated the touch screen UI as simple and less stressful. The authors attribute this result to the low user errors owing to the better visual-spatial assignment, low mode changes, and low physical strain compared with conventional operation using joystick and shift key.

Graphical UIs provide a promising approach as they allow to present different control levels simultaneously, and thus make them more easily accessible. Moreover, they provide a wide range of visualization opportunities to make the control characteristics more comprehensible. At the same time, most of the tablet-based UIs offer the possibility to connect additional input devices such as head or eye control. Sunny et al [31] also followed such an approach. The authors investigated the usability of a control system consisting of an eye-gaze interface and a tablet-based graphical UI for a wheelchair-mounted xArm 6 from UFactory in different manipulation tasks. A total of 10 healthy participants were included in the study. Although this is not a representative sample for the addressed target group of people with disabilities, high success rate could be achieved in the manipulation tasks. The participants highlighted the large buttons of the graphical UI as a key feature of usability in the design of the control system.

#### Requirements for Everyday Use

Consistent with the current state of the art on assistive robots, the results of our study demonstrate that a major challenge lies in the identification and classification of relevant task domains and associated motion and performance parameters. A key task here is to develop a taxonomy that balances robotic capabilities with health care requirements and user-centered needs.

In this context, research and development of assistive robots often refer to the International Classification of Functioning, Disability, and Health (ICF) [16,17]. The ICF is a standardized and international classification system for describing a person’s functional health status, disability, social impairment, and relevant environmental factors. For this purpose, the ICF is divided into two parts, each with 2 components: first, functioning and disability (components: body functions and structures, activities, and participation) and second, contextual factors (components: environmental factors and person-related factors). Each component is divided into different domains, which in turn are composed of different categories that form the units of the classification.

Thus, the ICF provides a standardized framework for classifying health-related functional limitations or requirements in the performance of ADLs and social participation. For robotic research and development, the ICF classification provides an important approach for identifying and developing functional parameters for robotic assistance and evaluating their performance. However, at the same time, the ICF does not provide a basis for identifying all tasks or making conclusions about their relevance and frequency in the everyday life of the individuals concerned. Therefore, more advanced approaches for the identification of relevant assistive activities and functional parameters are needed. Petrich et al [16] proposed such an approach. In their study, the authors investigated different lifelogging databases to determine both the frequency of ADL tasks in daily life and short-term arm and hand movements during domestic tasks.

Furthermore, in the process of prioritizing ADLs for robotic support, it is essential to consider the perspectives of third parties in the caring network. These parties play a crucial role because they form a secondary user group that will be involved in facilitating and supporting the use of assistive robots by the primary target group of people with functional limitations. Therefore, the consideration of their needs plays an essential role in acceptance and long-term use; however, the rating of ADL tasks varies between those parties [17].

### Limitations

The generalizability of the study results is subject to several limitations, which are discussed in this section. Owing to the small sample size, the results should be considered as indicative of future studies. In addition, several influencing factors were derived from the experimental and exploratory study design. The first factor is the safety measures taken owing to the early stage of technical development of the study demonstrator. Some of our findings suggest that these measures had an impact on the user evaluations (*scratching* scenario). In addition, the fact that we used a stationary robot from the industrial environment can be considered as another influencing factor (robot size). Current systems, such as the Kinova or Exact Dynamics systems, can be mounted on the user’s wheelchair and are characterized by a slim and lightweight design. Owing to the explorative pilot nature of the study, various influencing variables such as learning effect, novelty effect, and Hawthorne effect cannot be excluded. In this context, the study duration of 1 visit per participant should be mentioned as a particular factor. Therefore, the results presented in this paper need to be evaluated through further studies with field trials.

### Conclusions

Assistive robots are expected to have great potential in supporting and promoting the autonomy and independence of people with functional impairments in various ADLs. To achieve effective and efficient compensation of disease-related functional losses, high demands are imposed on user-friendly system design. In this context, this study investigated and discussed the requirements and needs of people with ALS for the development of a semiautonomous robotic manipulator for everyday life support. We identified 3 key requirement areas that should be pursued as foci of user-centered development in future research and development projects, consistent with previous studies. An essential prerequisite for development is the active and continuous involvement of the target group in the control processes. Therefore, a promising approach consists of adaptive semiautonomous control systems that enable the user to be involved in the autonomous decision-making and operational processes. A key question to be addressed is how to effectively mediate between the user’s skill level and the technical challenges in motion planning and object and environment recognition for efficient task accomplishment. Another focus of development is the UI. Owing to physical limitations, conventional input devices can be a high mental and physical burden in everyday life. Tablet-based graphical UIs can provide great relief in this regard, by simplifying access to various robot functions and making robot behavior more predictable and comprehensible through the use of diverse visualization options. Finally, there is a strong need to develop a specific taxonomy for assistive robots that provides a standardized assessment of task parameters, efficiency, and performance to serve as a comparative standard in research and development.

## Abbreviations

ADL: activities of daily living
ALS: amyotrophic lateral sclerosis
ALS-FRS: amyotrophic lateral sclerosis functional rating scale
GUI: graphical user input
ICF: International Classification of Functioning, Disability, and Health
RGB-D: red, green, blue, and depth
ROBINA: robot-assisted services for individual and resource-oriented intensive and palliative care of people with amyotrophic lateral sclerosis
SUS: System Usability Scale
UI: user interface
